# The Acute Effects of High-Intensity Interval Training on Oxidative Stress Markers and Phagocyte Oxidative Burst Activity in Young Professional Athletes and Non-Athlete University Students

**DOI:** 10.3390/life16010084

**Published:** 2026-01-06

**Authors:** László Balogh, Eszter Szklenár, Ádám Diós, Attila Csaba Arany, József Márton Pucsok, Zalán Mihály Bács, László Rátgéber, Zoltán Csiki, Ágnes Gyetvai, Gábor Papp

**Affiliations:** 1 Institute of Sport Sciences, University of Debrecen, 4032 Debrecen, Hungary; balogh.laszlo@sport.unideb.hu (L.B.); szklenar.eszter@gmail.com (E.S.); aranyattila4@gmail.com (A.C.A.); pucsok.jozsef@sport.unideb.hu (J.M.P.); bacs.zalan.m@gmail.com (Z.M.B.); 2 Division of Clinical Immunology, Institute of Internal Medicine, Faculty of Medicine, University of Debrecen, 4032 Debrecen, Hungary; dios.adam@med.unideb.hu (Á.D.); csiki@med.unideb.hu (Z.C.); gyetvaia@med.unideb.hu (Á.G.); 3 Institute of Physiotherapy and Sport Science, Faculty of Health Sciences, University of Pécs, 7621 Pécs, Hungary; ratgeber@ratgeber.hu

**Keywords:** high-intensity interval training, oxidative stress, antioxidant enzymes, phagocyte oxidative burst, professional athletes

## Abstract

During exercise, increased oxygen consumption results in elevated production of reactive oxygen species (ROS). If the antioxidant system is unable to counteract this surge in ROS, oxidative stress occurs. Physical activity modulates both the generation and clearance of ROS through dynamic interactions between metabolic and antioxidant systems, and also influences the oxidative burst activity of phagocytes, a key component of the innate immune response. To investigate the acute physiological responses to high-intensity interval training (HIIT), we assessed the effects of a single HIIT session on oxidative stress markers and the oxidative burst activity of phagocytes in young professional athletes and non-athlete individuals. Blood samples were collected before and after a HIIT session from eleven male athletes (mean age: 22.1 ± 4.5 years) and ten male non-athlete university students (mean age: 21.6 ± 2.3 years). Participants performed a single treadmill HIIT session of ten 45-s intervals at 75–85% of heart rate reserve, separated by 45-s low-intensity recovery periods, with target intensities individualized using the Karvonen formula. Total antioxidant capacity, activities of catalase, superoxide dismutase and glutathione peroxidase enzymes, total serum nitrite/nitrate levels, lipid peroxidation products, and oxidative burst activity of phagocytes were evaluated before and after exercise. In athletes, a significant increase was observed in the activity of superoxide dismutase (from a median of 2.09 to 2.21 U/mL; *p* = 0.037) and catalase (from a median of 32.94 to 45.45 nmol/min/mL; *p* = 0.034) after exercise, whereas no significant changes were found in the control group. Total serum nitrite/nitrate levels significantly increased in both groups after exercise (athletes: from a median of 8.70 to 9.95 µM; *p* = 0.029; controls: from a median of 10.20 to 11.50 µM; *p* = 0.016). Oxidative burst capacity of peripheral blood phagocytes was significantly higher in athletes both before (median: 10,422 vs. 6766; *p* = 0.029) and after (median: 9365 vs. 7370; *p* = 0.047) the HIIT session compared to controls. Our findings demonstrate that training status markedly influences oxidative stress responses, with athletes exhibiting more effective long-term antioxidant adaptations. These results emphasize the necessity of tailoring exercise regimens to baseline fitness levels in order to optimize oxidative stress management across different populations.

## 1. Introduction

Exercise is a form of physical activity that is planned, structured, repetitive, and purposefully focused on improving or maintaining one or more components of physical fitness [1]. Endurance training enhances resistance to fatigue and improves metabolic efficiency, while strength training primarily induces muscle hypertrophy and increased muscle strength. In recent decades, several new training methods have emerged, each influencing the human body in distinct ways [2]. Interval training alternates periods of intense effort with rest in a structured manner. Properly designed workouts can optimize health outcomes, including improvements in strength, endurance, and overall physiological adaptations [3]. High-intensity interval training (HIIT) is characterized by repeated bouts of exercise performed at 80–100% of maximum heart rate (HRmax), with intervals typically lasting from 6 s to 4 min, followed by short periods of reduced metabolic demand [4]. HIIT protocols can be individualized based on clinical status and physical capacity, making them suitable even for patients with coronary heart disease or chronic heart failure [5]. Since regular HIIT induces classical physiological adaptations, such as mitochondrial biogenesis and improvements in aerobic capacity (VO_2_max), it is considered one of the most effective methods for improving cardiorespiratory and metabolic functions [3,6,7].

Nevertheless, as an acute effect of exercise, increased oxygen consumption and muscle contraction can lead to the generation of reactive oxygen species (ROS). Exercise induces the activation of enzymes, such as NADPH oxidase, xanthine oxidase and phospholipase A2, which are known sources of ROS within the muscle. Additionally, muscle contraction leads to increased electron leakage from the mitochondrial electron transport chain, which subsequently results in increased superoxide formation [8]. Furthermore, oxidative burst activation of neutrophils and monocytes/macrophages provides another important source of ROS both in muscle tissue and in peripheral blood [9,10]. During intensive physical exercise, the production of free radicals may exceed the antioxidant capacity, which leads to the oxidation of biomolecules, such as proteins, lipids and nucleic acids [11]. In vivo oxidation of biomolecules has both detrimental and adaptive consequences. On one hand, it damages the biomolecules, causing their loss of function and degradation [12]. The antioxidant defense system can limit these undesirable consequences by enzymatic and non-enzymatic factors. The main antioxidant enzymes are catalase (CAT), superoxide dismutase (SOD) and glutathione peroxidase (GPx), while non-enzymatic antioxidants are glutathione, uric acid, bilirubin, ubiquinone and dietary antioxidants, including vitamin C, vitamin E and carotenoids [13]. On the other hand, oxidation is an important part of the physiological signaling process leading to beneficial adaptations, such as increased antioxidant enzyme expression, mitochondrial biogenesis, support of anabolic pathways, which contributes to increased fitness level [14,15,16].

However, exercise is a complex biological process that affects not only ROS production but also its elimination. The intensity and duration of exercise and the individual’s fitness level determine its effects on oxidative-antioxidant balance as well as oxidative burst activity of neutrophils and monocytes [10,17]. Nevertheless, the effects of HIIT on oxidative stress regulation are not yet fully elucidated; whereas certain studies demonstrate favorable antioxidant adaptations, others suggest the potential for enhanced pro-oxidant activity [18,19,20]. Considering these aspects, it is important to address how acute HIIT affects the dynamic balance between oxidants and antioxidant defense system, as well as the oxidative burst activity of neutrophils and monocytes, key components of the innate immune system, across different fitness levels. Therefore, this study aimed to investigate the acute effects of a single HIIT session on oxidative stress markers, antioxidant responses, and phagocyte oxidative burst activity in young professional athletes and non-athlete university students. In order to comprehensively assess the redox status and innate immune response, we selected a panel of physiologically relevant biomarkers, including SOD, CAT, and GPx, which are key markers of enzymatic antioxidant defenses. Total antioxidant capacity provided an integrated measure of non-enzymatic antioxidants, thiobarbituric acid reactive substances (TBARS) served as an indicator of lipid peroxidation and oxidative damage, while serum nitrite/nitrate levels were included as markers of nitric oxide metabolism. We also evaluated the ROS production capacity of peripheral blood phagocytes. These biomarkers served for a multidimensional characterization of oxidative stress and antioxidant responses to acute high-intensity exercise.

## 2. Materials and Methods

### 2.1. Participants

Eleven Hungarian male professional football players (mean age: 22.1 ± 4.5 years, mean BMI: 22.96 ± 2.46) and ten male untrained university students (mean age: 21.6 ± 2.3 years; mean BMI: 23.28 ± 4.12) as control subjects were enrolled in this study. Based on the training classification system developed by McKay et al. [21], the professional football players were categorized as Tier 3 (highly trained individuals), participating in regular training sessions (6 × 90 min per week) and weekly competitive matches. In contrast, the university students were classified as Tier 1 (recreationally active individuals), engaging in approximately 2 × 90 min of exercise per week. Participants were instructed to avoid strenuous physical activity for the previous 24 h and special diets or antioxidant supplementation for at least six weeks prior to the study. Moreover, exclusion criteria for participation included smoking, alcohol or drug addiction, ongoing viral or bacterial infection or chronic disease treated with continuous drug therapy. These criteria were evaluated through a structured medical interview conducted by a physician prior to the study.

### 2.2. Exercise Protocol

Resting heart rate (HRrest) was determined using a Polar Wrist Heart Rate Monitor (POLAR RS 300X, Polar Electro, Kempele, Finland) following a 10-min supine relaxation. HRmax was determined using the 220-age equation. Although the 220-age formula has known limitations, it was used here for consistency and feasibility across participants. Training heart rate zones corresponding to 60%, 70%, 80%, and 85% of heart rate reserve were calculated using the Karvonen formula: Target Heart Rate (THR) = ((HRmax − HRrest) × %Intensity) + HRrest. All HIIT sessions were conducted between 8:00 and 10:00 a.m., with each participant completing a single session for study purposes. After a standardized 10-min warm-up, participants completed a high-intensity interval training routine on Polar-compatible treadmills (STAR TRAC S-TRC, Star Trac, Irvine, CA, USA). The exercise routine included ten cycles of 45-s exercise sessions and 45-s active recovery periods. Target intensities gradually increased from 75 percent to 85 percent of maximal effort. The treadmill speed was manually adjusted to achieve the prescribed heart rate zones. Active recovery periods were performed as treadmill walking at a low intensity with treadmill speed set to 4 km/h (0% incline).

Table 1 demonstrates the resting heart rates and the target heart rates of training zones in the studied groups.

### 2.3. Blood Sampling and Analysis of Blood Cell Counts

In order to minimize both time-of-day variability and acute dietary influences on the investigated markers, all exercise sessions and blood sample collections were performed between 8:00 and 10:00 a.m., under standardized fasting conditions after an overnight fast. Blood samples were obtained from a forearm vein in a seated position before and immediately after the HIIT exercise (within 1–3 min) into VACUETTE^®^ CAT Serum Separator Tubes, VACUETTE^®^ EDTA Tubes and VACUETTE^®^ NH Sodium Heparin Tubes (Greiner Bio-One, Kremsmünster, Austria). Whole blood was centrifuged at 1000× *g* for 10 min, and the resulting samples were stored at −80 °C until analysis. Blood cell counts, including total neutrophil granulocyte counts, were analyzed from blood samples anticoagulated with ethylenediamine tetra-acetic acid (EDTA) with Sysmex XN-2000 Hematology Analyzer (Sysmex Europe GmbH, Norderstedt, Germany).

### 2.4. Quantification of the Oxidative Stress Biomarkers

The following oxidative stress biomarkers were evaluated: total antioxidant capacity, TBARS, total nitrite/nitrate levels, as well as the enzymatic activities of CAT, SOD and GPx. CAT and SOD activities, along with total antioxidant capacity, TBARS, and nitrite/nitrate levels, were measured from serum samples, while GPx activity was assessed from plasma. CAT activity was assessed using the Catalase Assay Kit (Cat. No. 707002) by quantifying formaldehyde production following hydrogen peroxide decomposition, using a colorimetric reaction with purpald. SOD activity was assessed with the Superoxide Dismutase Assay Kit (Cat. No. 706002), based on the enzyme’s capacity to neutralize superoxide radicals generated by xanthine oxidase. GPx activity was measured using the Glutathione Peroxidase Assay Kit (Cat. No. 703102), which indirectly quantifies enzyme activity by monitoring the consumption of NADPH in a glutathione reductase-catalyzed reaction. Total nitrite/nitrate concentrations were measured using the Nitrate/Nitrite Colorimetric Assay Kit (Cat. No. 780001). In this assay, serum nitrate is enzymatically converted to nitrite, which then reacts with Griess reagents to form a chromophore complex detectable by spectrophotometry. Total antioxidant capacity was assessed using the Antioxidant Assay Kit (Cat. No. 709001), which measures both enzymatic and non-enzymatic antioxidant components of the serum and expresses results in Trolox equivalents. Lipid peroxidation was quantified using the TBARS (TCA Method) Assay Kit (Cat. No. 700870), based on the formation of a colored complex between malondialdehyde (MDA) and thiobarbituric acid under acidic conditions. The kits were purchased from Cayman chemicals (Ann Arbor, MI, USA) and the assays were performed according to the instructions of the manufacturer using Labsystems Multiskan MS Type 352 microplate reader (Thermo Fisher Scientific, Waltham, MA, USA).

### 2.5. Measuring Oxidative Burst Activity of Phagocytes

Oxidative burst activity of the phagocytes was measured by Berthold Automat Plus BL 953 (Berthold, Bad Wildbad, Germany) luminometer based on the method previously described [22]. Oxidative burst was induced by zymosan particles (Merck KGaA, Darmstadt, Germany). Phosphate buffered saline (Merck KGaA, Darmstadt, Germany) was applied as negative control and phorbol 12-myristate 13-acetate (PMA, Merck KGaA, Darmstadt, Germany) as positive control. The oxidation of the luminol dye was followed for an hour by detecting the emitted photons (360–630 nm) at room temperature in every 5 min for 60 min. The chemiluminescence data were expressed in “Relative Light Units” (RLU). The oxidative burst activity was interpreted by comparing the area under the curve (AUC) values of the kinetic curves normalized to 1000 neutrophil cells in each sample.

### 2.6. Statistical Analysis

Statistical analyses were carried out with GraphPad Prism version 7.0 software (GraphPad Software, San Diego, CA, USA). Descriptive data were presented as box plots indicating the interquartile range (IQR), with the central line representing the median. Shapiro–Wilk normality tests were used to assess the distribution of the data. Differences between the parameters of professional athletes and control individuals were determined by unpaired two-sample *t*-test in case of normal distribution, while if the data set differed from normal distribution, Mann–Whitney test was used. For within-group comparisons of pre- and post-exercise values, paired *t*-test was applied under normal distribution, whereas Wilcoxon test was used for non-normally distributed data. Effect sizes were expressed as Cohen’s *d*, and differences were considered statistically significant at *p* < 0.05. Based on post hoc power analysis conducted in G*Power 3.1.9.7 (Heinrich-Heine-Universität Düsseldorf, Germany; α = 0.05, two-tailed, assumed large effect size d/dz = 0.80; paired tests: N = 21; unpaired tests: n_1_ = 10, n_2_ = 11), the paired *t*-test achieved a power of 0.94, the unpaired *t*-test a power of 0.41, the Wilcoxon test a power of 0.92, and the Mann–Whitney test a power of 0.40.

## 3. Results

### 3.1. Enzymatic Antioxidant Activities

**Figure 1:**
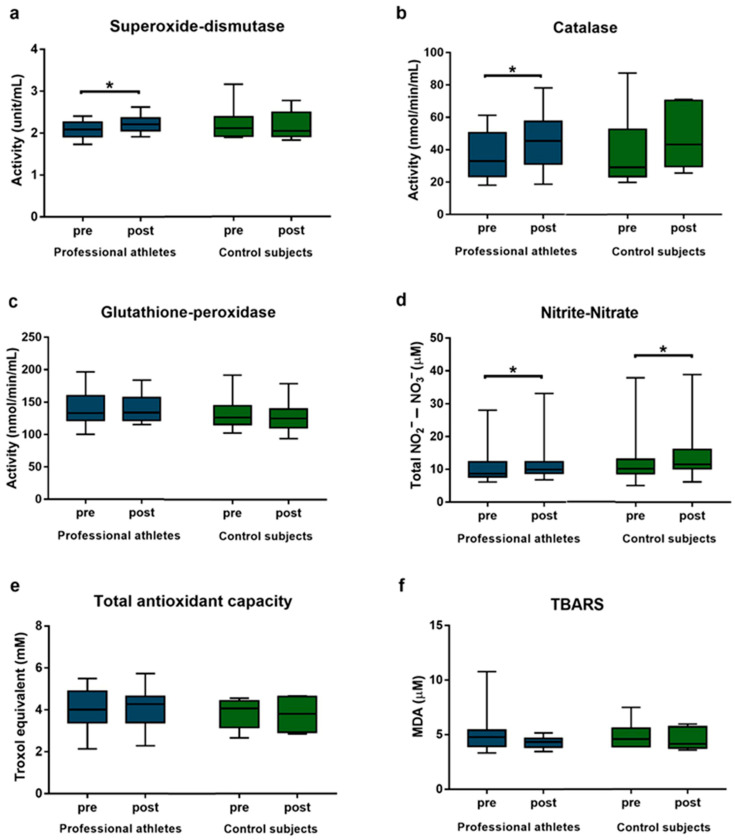
Antioxidant and oxidative stress markers in the peripheral blood of professional athletes (n = 11) and control subjects (n = 10) before and after an acute HIIT session. Panels (**a**–**f**) show SOD, CAT, GPx, nitrite/nitrate, total antioxidant capacity and TBARS levels respectively. Boxes represent interquartile ranges, horizontal lines show median values, and whiskers show minimum and maximum values. Statistically significant differences are indicated by * *p* < 0.05.

No significant differences were observed in baseline values between professional athletes and control participants. The antioxidant enzyme activities in the control group did not show any changes following the HIIT session. In contrast, the activity of SOD enzyme in professional athletes increased significantly following acute exercise, from a median of 2.09 U/mL (interquartile range [IQR]: 1.91–2.26 U/mL) to 2.21 U/mL (IQR: 2.06–2.36 U/mL; respectively, *p* = 0.037, *d* = 0.664). Similarly, CAT activity showed a significant post-exercise increase, from a median of 32.94 nmol/min/mL (IQR: 23.52–50.48 nmol/min/mL) to 45.45 nmol/min/mL (IQR: 31.09–57.55 nmol/min/mL; respectively, *p* = 0.034, *d* = 0.563) in professional athletes, while the activity of GPx did not change significantly (Figure 1a–c).

### 3.2. Nitrite and Nitrate Levels

Total nitrite/nitrate levels increased significantly in both groups. In athletes, levels rose from a median of 8.70 µM (IQR: 7.70–12.30 µM) to 9.95 µM (IQR: 8.80–12.30 µM; respectively, *p* = 0.029, *d* = 0.177). In the control group, levels increased from a median of 10.20 µM (IQR: 8.70–13.10 µM) to 11.50 µM (IQR: 10.20–16.10 µM; respectively, *p* = 0.016, *d* = 0.124) (Figure 1d).

### 3.3. Total Antioxidant Capacity and Thiobarbituric Acid Reactive Substances

Total antioxidant capacity and TBARS levels did not change significantly in either group following the HIIT session (Figure 1e,f).

### 3.4. Oxidative Burst Activity of Phagocytes

**Figure 2:**
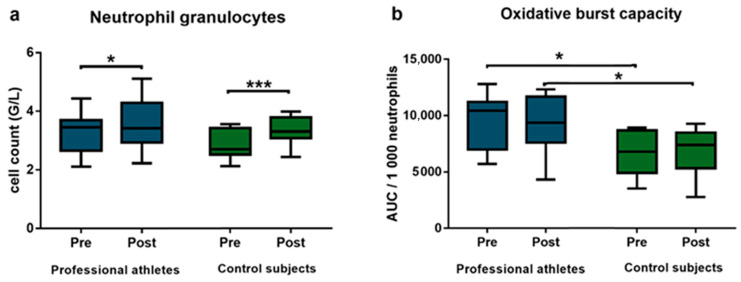
Neutrophil granulocyte counts (**a**) and zymosan-induced oxidative burst response (**b**) in professional athletes (n = 11) and control subjects (n = 10) before and after an acute HIIT session. Boxes represent interquartile ranges, horizontal lines show median values, and whiskers show minimum and maximum values. Statistically significant differences are indicated by * *p* < 0.05; *** *p* < 0.001.

In response to the HIIT session, we detected an increased neutrophil granulocyte cell count in the peripheral blood samples of both professional athletes (pre-HIIT median: 3.45 G/L; [IQR: 2.65–3.69 G/L] vs. post-HIIT median: 3.42 G/L; [IQR: 2.93–4.29 G/L]; respectively, *p* = 0.037, *d* = 0.420) and control subjects (pre-HIIT median: 2.70 G/L; [IQR: 2.52–3.43 G/L] vs. post-HIIT median: 3.31 G/L; [IQR: 3.08–3.79 G/L]; respectively, *p* = 0.0003, *d* = 0.993) (Figure 2a). The number of other leukocyte subsets showed no differences between the two groups and did not exhibit significant changes following the HIIT session.

When comparing the oxidative burst capacity of peripheral phagocytes, professional athletes exhibited significantly higher capacity both before (median: 10,422; [IQR: 6975–11,202]) and after (median: 9365; [IQR: 7582–11,686]) the HIIT session, compared to control subjects (pre-HIIT median: 6766; [IQR: 4896–8661]; respectively, *p* = 0.029, *d* = 1.229 and post-HIIT median: 7370; [IQR: 5310–8470]; respectively, *p* = 0.047, *d* = 1.094). However, the oxidative burst capacity did not change significantly when comparing pre- and post-HIIT values within either group (Figure 2b).

## 4. Discussion

Among contemporary training methods, HIIT has received particular attention in recent years due to its time efficiency and physiological effectiveness. Growing evidence suggests that HIIT has beneficial effects on various health parameters, including cardiovascular functions, low-grade inflammation and quality of life [23,24,25,26]. Despite requiring less total time, HIIT has been reported to elicit comparable or even greater improvements in cardiometabolic parameters relative to moderate-intensity continuous aerobic training. Furthermore, evidence supports its safety and feasibility across diverse patient populations [4,27]. Beyond the well-documented improvements in cardiorespiratory fitness, novel studies indicate that exercise-induced adaptation of the antioxidant defense system constitutes a central mechanism underlying enhanced cardiometabolic health [28].

High-intensity phases of HIIT acutely enhance ROS generation, promoting a transient state of oxidative stress. Notably, this rise reflects a normal physiological reaction to heightened metabolic demands and mitochondrial activity, alongside increased activity of ROS-producing enzymes, including nicotinamide adenine dinucleotide phosphate (NADPH) oxidase (NOX) and uncoupled nitric oxide synthase [19]. During intense exercise, multiple tissues, particularly skeletal muscle and the vascular endothelium experience elevated oxygen flux, resulting in increased electron leakage from the mitochondrial electron transport chain and consequent amplification of superoxide production [29]. This acute surge in ROS is not inherently pathological; rather, it functions as a physiological signal. This process, commonly referred to as “oxidative eustress”, triggers various signaling pathways, including the NF-κB and nuclear factor erythroid 2–related factor 2 (Nrf2) transcription factors, which in turn upregulate the synthesis of endogenous antioxidants, such as SOD, CAT, and GPx [19]. The extent of this acute oxidative response is largely determined by the intensity and duration of the exercise, as higher intensities and longer training sessions can amplify oxidative stress, potentially exceeding the capacity of endogenous antioxidant systems [30]. By design, HIIT comprises brief bouts of vigorous exercise, typically performed at 85–95% of HRmax. Such intensities can induce substantial acute oxidative stress; however, the relatively low total exercise volume in short HIIT protocols may restrict the period of oxidative stress, thereby reducing the risk of substantial oxidative damage relative to longer-duration endurance training. Importantly, these mechanisms can also be modulated by an individual’s training status and overall health. In sedentary populations, even moderate-to-vigorous exercise may trigger a relatively greater increase in oxidative stress markers, likely reflecting their lower baseline antioxidant capacity. By contrast, individuals with a high level of training can more effectively buffer the same oxidative challenge through prior upregulation of enzymatic and non-enzymatic antioxidant defenses [30]. Previous studies have demonstrated that regular exercise enhances both the activity and production of major antioxidant enzymes, including SOD, CAT, and GPx [31,32,33]. Such adaptations contribute to improved redox homeostasis and attenuated basal oxidative damage. Bogdanis et al. reported that following a short-term HIIT program, there was an increase in CAT activity in skeletal muscle in healthy men after only 3 weeks [34]. Costa et al. likewise reported that a 4-week progressive HIIT protocol significantly increased plasma total antioxidant capacity and erythrocyte CAT activity in healthy young men, reflecting improved systemic redox homeostasis [35]. In contrast, we observed no significant differences in baseline antioxidant parameters including SOD, CAT, GPx and total antioxidant capacity between young professional athletes and non-athlete university students in our study. However, we found that SOD and CAT activities were significantly elevated in professional athletes following HIIT, whereas university students did not exhibit comparable increases. Thereby, our findings still support the notion that regular training plays a pivotal role in the adaptation of antioxidant responses.

We also investigated the changes in total nitrite and nitrate levels after the HIIT session, which reflects the extent of nitric oxide (NO) production, a potent vasodilator and signaling molecule, during exercise [36]. NO plays a key role in exercise physiology by regulating vascular tone, muscle blood flow, and oxygen delivery, and also supports mitochondrial biogenesis and efficiency. Exercise promotes NO synthesis through enhanced endothelial nitric oxide synthase (eNOS) expression and phosphorylation, and increases NO bioavailability by reducing its deactivation, collectively leading to elevated plasma nitrite/nitrate levels, which is generally considered beneficial for vascular function and exercise performance [37]. Previous studies in older populations suggested that training status may positively influence basal nitrite levels, possibly compensating for the age-related decline in NO bioavailability [38,39]. However, we did not observe such differences in healthy young adults, as baseline values did not differ between athletes and controls. On the other hand, we observed that a single HIIT session acutely increased total nitrite/nitrate levels in both athletes and controls. Although this rise in total nitrite/nitrate levels is consistent with exercise-induced NO production, habitual dietary nitrate intake may also influence its resting levels. This factor may partially explain the lack of baseline differences between groups, despite their divergent training status. Regarding the extent of lipid peroxidation, no differences were observed between the two groups, and HIIT did not elicit any significant changes. A recent systematic review indicates that total antioxidant capacity and TBARS levels can change significantly immediately after exercise. Therefore, our findings may suggest that the antioxidant response to HIIT functioned appropriately in both professional athletes and healthy non-athlete controls; however, a delayed peak response—reported in other studies—cannot be ruled out [18]. Alternatively, it is also possible that the level of exercise-induced oxidative stress in our protocol did not exceed the threshold required to elicit a measurable increase in these biomarkers among the young participants.

We observed a significant elevation in neutrophil granulocyte cell count in both professional athletes and control subjects following a HIIT session, with the increase being more pronounced in the control group. This may be attributed to the athletes’ regular exposure to high-frequency training sessions, resulting in a reduced neutrophil mobilization response due to prior physiological adaptation from regular high-frequency training. Nevertheless, the HIIT protocol effectively promoted phagocyte mobilization from peripheral pools in both groups, potentially enhancing immunosurveillance and host defense mechanisms [40]. Since phagocytes are key components of the innate immune system and comprise the first line of defense against foreign pathogens, their bactericidal functions are crucial in effective immune responses. Exercise exerts systemic effects on immune function and inflammation. While some anti-inflammatory benefits of exercise may be mediated by changes in adipose tissue, cellular immune function also appears to be directly modulated [41,42]. A number of studies reported enhanced activity and phagocytosis of neutrophil granulocytes in the peripheral blood in relation to regular exercise [43,44], indicating enhanced ROS production capacity, which may translate to superior antimicrobial defense. In our study, although systemic oxidative stress and antioxidant markers showed no baseline differences between athletes and non-athletes, baseline oxidative burst capacity of phagocytes was substantially higher in athletes. This distinction highlights that innate immune cell function adapts to chronic training independently of systemic redox status, likely reflecting sport-related enhancement of NADPH oxidase–mediated ROS generation and immunological readiness [45,46,47]. Although high-intensity training has been reported to blunt phagocyte oxidative burst capacity and contribute to neutrophil exhaustion—potentially increasing infection susceptibility in professional athletes [48,49]—our findings indicate that the HIIT session applied in this study did not alter oxidative burst capacity within the immediate post-exercise window (1–3 min after exercise) in either group. Therefore, our conclusion that HIIT does not impair phagocytic oxidative function applies specifically to this acute time frame; delayed post-exercise effects cannot be assessed based on the current sampling protocol.

Finally, it is necessary to consider some limitations in this study. One important limitation is the single time-point assessment of antioxidant capacity parameters, which does not provide information about the dynamic changes of the measured parameters. However, strong evidence from a recent systematic review indicates that acute oxidative stress occurs upon cessation of high-intensity exercise, and measurable changes in oxidative markers typically occur immediately following exercise [18]. Although the exact kinetics of individual oxidative stress markers may vary depending on the population, exercise modality, and methodology, immediate post-exercise sampling is widely considered an appropriate and sensitive window for detecting rapid redox perturbations and initial antioxidant responses. Therefore, we selected this time point to capture the earliest measurable systemic changes induced by HIIT, in alignment with the main objectives of our study. Another main limitation is the modest sample size, which naturally reduces the statistical power for detecting smaller effect sizes and limits the generalizability of the findings. Nevertheless, the consistent response patterns observed across participants support the physiological relevance of the results, and the homogeneous characteristics of both groups strengthen the internal validity of the study. On the other hand, the study also has notable strengths, including the individualized and carefully controlled exercise protocol, as well as the comprehensive evaluation of a broad spectrum of antioxidant markers. Furthermore, by including only male participants, we were able to eliminate potential confounding effects associated with hormonal fluctuations across the menstrual cycle; however, this approach also limits the generalizability of our findings to female athletes and non-athletes. Finally, in our study, the relative intensity of the HIIT protocol was individualized using heart rate reserve, ensuring a physiologically comparable stimulus across participants. Nevertheless, future studies incorporating CPET [50] could provide additional insight into how precise aerobic fitness interacts with redox and immune responses, further refining our understanding of the relationship between chronic training status and acute physiological adaptations.

## 5. Conclusions

One single bout of HIIT induced clear antioxidant responses in trained athletes but not in untrained young adults, underscoring the role of regular physical activity in enhancing enzymatic antioxidant capacity. In contrast, nitrite/nitrate levels increased similarly in both groups, suggesting that this acute response is independent of training status. Athletes also exhibited consistently higher phagocyte oxidative burst capacity, indicating enhanced innate immune function associated with chronic training. Overall, our findings highlight that training status markedly shapes redox and immune responses to acute high-intensity exercise.

## Figures and Tables

**Table 1:** Resting and target heart rates of training zones.

	Professional Athletes (n = 11)	Control Subjects (n = 10)	*p* Value
Resting heart rate (mean ± SD)	57.4 ± 7.04	68 ± 6.22	0.003
Target heart rate for 60% intensity (mean ± SD)	142.1 ± 3.41	145.4 ± 2.14	0.028
Target heart rate for 70% intensity (mean ± SD)	156.2 ± 3.21	158.3 ± 1.60	0.119
Target heart rate for 80% intensity (mean ± SD)	170.3 ± 3.22	171.2 ± 1.24	0.498
Target heart rate for 85% intensity (mean ± SD)	177.4 ± 3.31	177.7 ± 1.20	0.840

## Data Availability

The original contributions presented in this study are included in the article; further inquiries can be directed to the corresponding author.

## References

[1] Dasso N.A. (2019). How is exercise different from physical activity? A concept analysis. Nurs. Forum.

[2] Balogh L., Szklenár E., Makra G., Torma E., Rátgéber L., Bíró E., Bács Z., Pucsok J.M., Papp G. (2025). Comparative analysis of ten-week high-intensity interval training, moderate-intensity continuous training, and proprioceptive workouts: Impact on cognitive abilities, body composition, perceived stress and motor skills. J. Phys. Educ. Sport.

[3] MacInnis M.J., Gibala M.J. (2017). Physiological adaptations to interval training and the role of exercise intensity. J. Physiol..

[4] Weston K.S., Wisløff U., Coombes J.S. (2014). High-intensity interval training in patients with lifestyle-induced cardiometabolic disease: A systematic review and metaanalysis. Br. J. Sports Med..

[5] Moholdt T., Madssen E., Rognmo Ø., Aamot I.L. (2014). The higher the better? Interval training intensity in coronary heart disease. J. Sci. Med. Sport.

[6] Alibrahim M.S., Hassan A.K. (2024). Effect of high-intensity interval training on physical and biological indicators in individual sports Athletes. J. Phys. Educ. Sport.

[7] Buchheit M., Laursen P. (2013). High-intensity interval training, solutions to the programming puzzle: Part I: Cardiopulmonary emphasis. Sports Med..

[8] Jackson M.J., Sen C.K., Packer L., Hanninen O. (2000). Exercise and oxygen radical production by muscle. Handbook of Oxidants and Antioxidants in Exercise.

[9] Baumert P., Hall E.C., Erskine R.M., Barh D., Ahmetov I.I. (2019). The genetic association with exercise-induced muscle damage and muscle injury risk. Sports, Exercise, and Nutritional Genomics (Current Status and Future Directions).

[10] Bartlett D.B., Shepherd S.O., Wilson O.J., Adlan A.M., Wagenmakers A.J.M., Shaw C.S., Lord J.M. (2017). Neutrophil and Monocyte Bactericidal Responses to 10 Weeks of Low-Volume High-Intensity Interval or Moderate-Intensity Continuous Training in Sedentary Adults. Oxid. Med. Cell. Longev..

[11] Dalle-Donne I., Rossi R., Colombo R., Giustarini D., Milzani A. (2006). Biomarkers of oxidative damage in human disease. Clin. Chem..

[12] Halliwell B., Gutteridge J.M.C. (2015). Free Radicals in Biology & Medicine.

[13] Powers S.K., Jackson M.J. (2008). Exercise-Induced Oxidative Stress: Cellular Mechanisms and Impact on Muscle Force Production. Physiol. Rev..

[14] Gomez-Cabrera M.C., Domenech E., Vina J. (2008). Moderate Exercise Is an Antioxidant: Upregulation of Antioxidant Genes by Training. Free Radic. Biol. Med..

[15] Ji L.L. (2008). Modulation of Skeletal Muscle Antioxidant Defense by Exercise: Role of Redox Signaling. Free Radic. Biol. Med..

[16] Powers S.K., Duarte J., Kavazis A.N., Talbert E.E. (2010). Reactive oxygen species are signalling molecules for skeletal muscle adaptation. Exp. Physiol..

[17] Radak Z., Ishihara K., Tekus E., Varga C., Posa A., Balogh L., Boldogh I., Koltai E. (2017). Exercise, oxidants, and antioxidants change the shape of the bell-shaped hormesis curve. Redox Biol..

[18] Lu Y., Wiltshire H.D., Baker J.S., Wang Q. (2021). Effects of High Intensity Exercise on Oxidative Stress and Antioxidant Status in Untrained Humans: A Systematic Review. Biology.

[19] Powers S.K., Deminice R., Ozdemir M., Yoshihara T., Bomkamp M.P., Hyatt H. (2020). Exercise-induced oxidative stress: Friend or foe?. J. Sport Health Sci..

[20] Thirupathi A., Wang M., Lin J.K., Fekete G., István B., Baker J.S., Gu Y. (2021). Effect of Different Exercise Modalities on Oxidative Stress: A Systematic Review. Biomed. Res. Int..

[21] McKay A.K.A., Stellingwerff T., Smith E.S., Martin D.T., Mujika I., Goosey-Tolfrey V.L., Sheppard J., Burke L.M. (2022). Defining Training and Performance Caliber: A Participant Classification Framework. Int. J. Sports Physiol. Perform..

[22] Szabó Z., Szilasi M., Brúgós L., Szántó S., Kovács I., Széles M., Lakos G., Antal-Szalmás P., Edes I., Sipka S. (2000). Differences in the changes of allergen-specific IgE serum levels and the chemiluminescence of peripheral blood phagocytes in patients with allergic rhinoconjunctivitis during the ragweed season. Immunol. Lett..

[23] Ko J.M., So W.Y., Park S.E. (2025). Narrative Review of High-Intensity Interval Training: Positive Impacts on Cardiovascular Health and Disease Prevention. J. Cardiovasc. Dev. Dis..

[24] Reljic D., Dieterich W., Herrmann H.J., Neurath M.F., Zopf Y. (2022). “HIIT the inflammation”: Comparative effects of low volume interval training and resistance exercises on inflammatory indices in obese metabolic syndrome patients undergoing caloric restriction. Nutrients.

[25] Yin M., Li H., Bai M., Liu H., Chen Z., Deng J., Deng S., Meng C., Vollaard N.B.J., Little J.P. (2024). Is low-volume highintensity interval training a time-efficient strategy to improve cardiometabolic health and body composition? A meta-analysis. Appl. Physiol. Nutr. Metab..

[26] Griffiths M., Edwards J.J., McNamara J., Galbraith S., Bruce-Low S., O’Driscoll J.M. (2024). The effects of high intensity interval training on quality of life: A systematic review and meta-analysis. J. Public Health.

[27] Francois M.E., Little J.P. (2015). Effectiveness and safety of high-intensity interval training in patients with type 2 diabetes. Diabetes Spectr..

[28] Meng Q., Su C.-H. (2024). The Impact of Physical Exercise on Oxidative and Nitrosative Stress: Balancing the Benefits and Risks. Antioxidants.

[29] Gomez-Cabrera M.C., Domenech E., Ji L.L., Viña J. (2006). Exercise as an antioxidant: It up-regulates important enzymes for cell adaptations to exercise. Sci. Sports.

[30] Lu Z., Song Y., Chen H., Li S., Teo E.C., Gu Y. (2022). A Mixed Comparisons of Aerobic Training with Different Volumes and Intensities of Physical Exercise in Patients with Hypertension: A Systematic Review and Network Meta-Analysis. Front. Cardiovasc. Med..

[31] Marin D.P., Bolin A.P., Campoio T.R., Guerra B.A., Otton R. (2013). Oxidative stress and antioxidant status response of handball athletes: Implications for sport training monitoring. Int. Immunopharmacol..

[32] Wiecek M., Szymura J., Maciejczyk M., Kantorowicz M., Szygula Z. (2018). Anaerobic Exercise-Induced Activation of Antioxidant Enzymes in the Blood of Women and Men. Front. Physiol..

[33] Bilici Ö.F., Erkan D., Alexe D.I., Tohănean D.I., Demir C., Alexe C.I., Voiculescu V.E., Bilici M.F., Fuentes-Barria H., Yildirim U.C. (2025). Biochemical Effects of Long-Term Exercise on Oxidative Stress and Antioxidant Markers in Adolescent Female Athletes. Children.

[34] Bogdanis G.C., Stavrinou P., Fatouros I.G., Philippou A., Chatzinikolaou A., Draganidis D., Ermidis G., Maridaki M. (2013). Short-term high-intensity interval exercise training attenuates oxidative stress responses and improves antioxidant status in healthy humans. Food Chem. Toxicol..

[35] Costa K.B., Magalhães S.M., Aguiar P.F., Ottone V.O., Tossige-Gomes R., Magalhães F.C., Amorim F.T., Rocha-Vieira E. (2018). Modification of Blood Redox Homeostasis by High-Intensity Interval Training. React. Oxyg. Species.

[36] Arefirad T., Seif E., Sepidarkish M., Mohammadian Khonsari N., Mousavifar S.A., Yazdani S., Rahimi F., Einollahi F., Heshmati J., Qorbani M. (2022). Effect of exercise training on nitric oxide and nitrate/nitrite (NOx) production: A systematic review and meta-analysis. Front. Physiol..

[37] Mueller B.J., Roberts M.D., Mobley C.B., Judd R.L., Kavazis A.N. (2025). Nitric oxide in exercise physiology: Past and present perspectives. Front. Physiol..

[38] Jacomini A.M., de Souza H.C., Dias Dda S., Brito Jde O., Pinheiro L.C., da Silva A.B., da Silva R.F., Trapé A.A., De Angelis K., Tanus-Santos J.E. (2015). Training Status as a Marker of the Relationship between Nitric Oxide, Oxidative Stress, and Blood Pressure in Older Adult Women. Oxid. Med. Cell. Longev..

[39] Trapé A.A., Jacomini A.M., Muniz J.J., Sertorio J.T., Tanus-Santos J.E., do Amaral S.L., Zago A.S. (2013). The relationship between training status, blood pressure and uric acid in adults and elderly. BMC Cardiovasc. Disord..

[40] Nieman D.C., Wentz L.M. (2019). The compelling link between physical activity and the body’s defense system. J. Sport Health Sci..

[41] Gleeson M., Bishop N.C., Stensel D.J., Lindley M.R., Mastana S.S., Nimmo M.A. (2011). The anti-inflammatory effects of exercise: Mechanisms and implications for the prevention and treatment of disease. Nat. Rev. Immunol..

[42] Papp G., Szabó K., Jámbor I., Mile M., Berki A.R., Arany A.C., Makra G., Szodoray P., Csiki Z., Balogh L. (2021). Regular Exercise May Restore Certain Age-Related Alterations of Adaptive Immunity and Rebalance Immune Regulation. Front. Immunol..

[43] Michishita R., Shono N., Inoue T., Tsuruta T., Node K. (2010). Effect of exercise therapy on monocyte and neutrophil counts in overweight women. Am. J. Med. Sci..

[44] Syu G.D., Chen H.I., Jen C.J. (2012). Differential effects of acute and chronic exercise on human neutrophil functions. Med. Sci. Sports Exerc..

[45] Sasaki S., Matsuura T., Takahashi R., Iwasa T., Watanabe H., Shirai K., Nakamoto H., Goto S., Akita S., Kobayashi Y. (2013). Effects of regular exercise on neutrophil functions, oxidative stress parameters and antibody responses against 4-hydroxy-2-nonenal adducts in middle aged humans. Exerc. Immunol. Rev..

[46] Giraldo E., Garcia J.J., HinChado M.D., Ortega E. (2009). Exercise intensity-dependent changes in the inflammatory response in sedentary women: Role of neuroendocrine parameters in the neutrophil phagocytic process and the pro-/anti-inflammatory cytokine balance. Neuroimmunomodulation.

[47] Levada-Pires A.C., Lambertucci R.H., Mohamad M., Hirabara S.M., Curi R., Pithon-Curi T.C. (2007). Exercise training raises expression of the cytosolic components of NADPH oxidase in rat neutrophils. Eur. J. Appl. Physiol..

[48] Pyne D.B., Smith J.A., Baker M.S., Telford R.D., Weidemann M.J. (2000). Neutrophil oxidative activity is differentially affected by exercise intensity and type. J. Sci. Med. Sport.

[49] Robson P.J., Blannin A.K., Walsh N.P., Castell L.M., Gleeson M. (1999). Effects of exercise intensity, duration and recovery on in vitro neutrophil function in male athletes. Int. J. Sports Med..

[50] Kasiak P., Kowalski T., Klusiewicz A., Zdanowicz R., Ładyga M., Wiecha S., Mamcarz A., Śliż D. (2024). Recalibrated FRIEND equation for peak oxygen pulse is accurate in endurance athletes: The NOODLE study. Sci. Rep..

